# Ancient bacteria of the Ötzi’s microbiome: a genomic tale from the Copper Age

**DOI:** 10.1186/s40168-016-0221-y

**Published:** 2017-01-17

**Authors:** Gabriele Andrea Lugli, Christian Milani, Leonardo Mancabelli, Francesca Turroni, Chiara Ferrario, Sabrina Duranti, Douwe van Sinderen, Marco Ventura

**Affiliations:** 1 Laboratory of Probiogenomics, Department of Life Sciences, University of Parma, Parco Area delle Scienze 11a, 43124 Parma, Italy; 2 APC Microbiome Institute and School of Microbiology, National University of Ireland, Cork, Ireland

**Keywords:** Metagenomics, Genomics, Genomic evolution, Gut bacteria

## Abstract

**Background:**

Ancient microbiota information represents an important resource to evaluate bacterial evolution and to explore the biological spread of infectious diseases in history. The soft tissue of frozen mummified humans, such as the Tyrolean Iceman, has been shown to contain bacterial DNA that is suitable for population profiling of the prehistoric bacteria that colonized such ancient human hosts.

**Results:**

Here, we performed a microbial cataloging of the distal gut microbiota of the Tyrolean Iceman, which highlights a predominant abundance of *Clostridium* and *Pseudomonas* species. Furthermore, in silico analyses allowed the reconstruction of the genome sequences of five ancient bacterial genomes, including apparent pathogenic ancestor strains of *Clostridium perfringens* and *Pseudomonas veronii* species present in the gut of the Tyrolean Iceman.

**Conclusions:**

Genomic analyses of the reconstructed *C. perfringens* chromosome clearly support the occurrence of a pathogenic profile consisting of virulence genes already existing in the ancient strain, thereby reinforcing the notion of a very early speciation of this taxon towards a pathogenic phenotype. In contrast, the evolutionary development of *P. veronii* appears to be characterized by the acquisition of antibiotic resistance genes in more recent times as well as an evolution towards an ecological niche outside of the (human) gastrointestinal tract.

**Electronic supplementary material:**

The online version of this article (doi:10.1186/s40168-016-0221-y) contains supplementary material, which is available to authorized users.

## Background

The (healthy) human gut harbors a highly complex and abundant microbial community (representing bacteria, viruses, and protozoa), also known as the gut microbiota, which exists in an equilibrium with its host [1]. The composition of such a microbiota is markedly influenced by the environment and diet, as well as host’s genetics and health status [2]. Since many of the microorganisms harbored by the gastrointestinal tract of animals are considered symbiotic, i.e., their presence is beneficial for both host and microbe, shifts in microbiota composition can exert a substantial impact on the host’s physiology. Moreover, disruption of the equilibrium between gut symbionts can lead to colonization of pathogens or overgrowth of opportunistic pathogens, thus causing infectious diseases [3, 4].

In recent years, metagenomic attempts have been carried out to delineate the composition of microbial communities from complex environments such as the human gut [5]. Using Next-generation sequencing (NGS) approaches, it has been possible to investigate the composition of the gut microbiota by sequencing the 16S rRNA marker gene of the bacteria residing in the biological sample, i.e., stool or mucosal biopsy, without the need for microbial cultivation [6–8]. Similar approaches have been applied to characterize the microbiota composition of ancient biological samples retrieved from mummies [9–11]. Several types of ancient specimens, including both coprolites as well as human remains, have been studied during recent years in order to elucidate their microbial associations [11, 12]. In this context, the oral cavity [13, 14] and distal gut microbiota have been extensively analyzed [9, 10]. Notably, depending on climate and storage conditions, genomic data retrieved from ancient biological samples may be inaccurate, due to post-mortem bacterial community alterations [11, 15]. Nevertheless, sufficiently low temperatures such as permafrost are believed to represent the optimal conditions for long-term DNA conservation of ancient DNA [16] and for the prevention of post-mortem shifts of bacterial community profiles [17]. In this context, it has recently been showed that when the remains of a deceased are kept at 4 °C, it will take more than 5 days before significant post-mortem bacterial community alterations are observed [18]. This finding suggests that the environmental temperature plays a key role in the stability of post-mortem gut microbiota composition.

Ancient microbiota data sets represent an important source of information that may facilitate the reconstruction of bacterial evolution. Furthermore, collected ancient microbial DNA information may be employed to explore the biological causes and frequency of infectious diseases in history as recently performed for the identification of ancient bacterial pathogens in mummified humans [19–21]. Finally, in recent years, the combination of DNA enrichment methodologies and NGS has enabled researchers to completely reconstruct ancient genomes like those of the pathogens *Yersinia pestis* [19], *Mycobacterium leprae* [20], and *Helicobacter pylori* [21].

The best known frozen and mummified human body, called Ötzi, also referred to as the Tyrolean Iceman, was found in an Italian Alpine glacier [22]. The well-preserved body of Ötzi allowed the retrieval of biological samples from various anatomical regions of this ancient human being [23–25]. A first insight into Ötzi’s microbiota composition was obtained from his stomach and colon contents [10]. Recently, an accurate screening of the stomach samples allowed the reconstruction of the genome of the pathogen *H. pylori*, while it also permitted the study of its relatedness to modern *H. pylori* strains retrieved from around the globe [21].

In this study, we performed an in depth metagenomic analysis based on data derived from four biopsy samples recently retrieved from the small and large intestines of the Tyrolean Iceman [21], in an attempt to reconstruct the dominant microbial genomes that constitute the Tyrolean Iceman’s distal gut microbiome.

## Methods

### Genome sequences and metagenome samples

We retrieved complete and partial genome sequences of 20 *Clostridium* and 90 *Pseudomonas* strains from the National Center for Biotechnology Information (NCBI) public database (Additional file 1: Table S1). Illumina HiSeq 2000 paired-end sequencing data of the Tyrolean Iceman gut were retrieved from the European Nucleotide Archive under accession ERP012908 (Additional file 1: Table S2).

### Ancient DNA extraction and Illumina libraries preparation

Analyses were performed involving DNA samples processed at the “ancient DNA” Laboratory of the EURAC-Institute for Mummies and the Iceman, Bolzano, Italy as previously described [21]. Sample preparation and DNA extraction were performed in a dedicated pre-PCR area following the strict procedures required for studies of ancient DNA, which involved the use of protective clothing, UV-light exposure of the equipment and bleach sterilization of surfaces, use of PCR workstations, and filtered pipette tips. DNA extraction was performed with approximately 40 mg of stomach mucosa tissue and 250 mg of gastrointestinal tract content samples using a chloroform-based DNA extraction method according to the protocol of Tang et al. [26]. Negative controls for all experimental steps were included to rule out contamination. DNA was extracted from 100 mg of soft tissue by a magnetic bead-based technology using the Biorobot®-EZ1 (Qiagen, Hilden, Germany), following a previously described procedure [27].

Library preparation and sequencing were performed in DNA-free benches in separate rooms dedicated to aDNA procedures at Kiel University. Libraries for the Illumina runs with the IDs A1140, A1141, A1142, A1144, A1145, and A1146 were prepared from 50 μl of each DNA extract using the Truseq Kit v2.0 (Illumina) and the adapters AD001-AD012, following the manufacturer’s protocol. For all purification steps, the Qiaquick Kit (Qiagen, Hilden, Germany) was applied according to the manufacturer’s protocol.

Libraries for the sequencing runs were generated from 20 μl of each aDNA extract applying a modified protocol for Illumina multiplex sequencing [28, 29]. For the samples as well as all extraction and library blank controls, unique indexes were added to both library adapters [28]. A second amplification was performed for all indexed libraries in a 50-μl reaction containing 5 μl library template, 2 U AccuPrime Pfx DNA polymerase (Invitrogen), 1 U 10× PCR Mix and 0.3 μM of each primer IS5 and IS6 [29]. The following thermal profile was used: a 2-min initial denaturation at 95 °C, 3, 4, or 8 cycles consisting of 15 s denaturation at 95 °C, a 30-s annealing at 60 °C and a 2-min elongation at 68 °C, and as a final step at the end of the cycles a 5-min elongation at 68 °C. The amplified libraries were purified using the Qiaquick Kit (Qiagen, Hilden, Germany). Subsequently, the sequencing libraries were quantified with the Agilent 2100 Bioanalyzer DNA 1000 chip. The sequencing was carried out on the Illumina HiSeq 2000 and 2500 platform at the Institute of Clinical Molecular Biology, Kiel University, by 2 × 101 cycles using the HiSeq v3 chemistry and the manufacturer’s protocol for multiplex sequencing.

### Metagenome assemblies

Fastq files of the paired-end reads obtained from shotgun sequencing of the Tyrolean Iceman’s small and large intestines, i.e., B0625 (lower part of the large intestine), C1824 and C1825 (upper part of the large intestine), and B0621 (small intestine) (Additional file 1: Table S2) were used as input for the genome assemblies through the MEGAnnotator pipeline [30]. The SPAdes program (version 3.5) was used for de novo metagenomic assemblies [31].

### Ancient DNA pattern identification

The mapped reads obtained from a selection of the most abundant bacterial species colonizing the Ötzi’s gut were used to confirm their belonging to ancient microorganisms. The selected reads were mapped to their reference genomes (Table 1) in order to assess possible nucleotide mis-incorporation patterns along the DNA fragments. The resulting bam files from the read alignments were checked for ancient DNA patterns using mapDamage [32] in order to depict the C to T mis-incorporation pattern at the 5′ end of the reads.

**Table 1:** Ancient genome features

Species	Strain	Genome size	ORFs number	GC content	Contigs	COGs^a^	Core genes^a^	Unique genes^a^	ANI^b^	Related strain
*Clostridium* sp.	CADE	2,713,669	2605	27.06	333	3,119	1,993	215	90.04	*Clostridium* sp. Ade.TY
*Clostridium algidicarnis*	CALG	2,793,371	2588	29.98	95	2,902	2,295	216	98.38	*Clostridium algidicarnis* B3
*Clostridium perfringens*	CPER	3,860,191	3739	27.72	1,018	7,720	1,737	493	98.46	*Clostridium perfringens* SM101
*Pseudomonas fluorescens*	PFLU	5,806,496	5839	59.05	1,486	33,938	1,627	785	87.4	*Pseudomonas fluorescens* H14
*Pseudomonas syringae*	PSYR	2,744,354	2771	57.44	642	–	–	–	–	–
*Pseudomonas veronii*	PVER	6,299,241	5805	60.49	289	8,700	4,200	615	93.89	*Pseudomonas veronii* R4

^a^Data obtained through pan-genome analyses with the public available sequenced genomes of the same species

^b^Average nucleotide identity of the whole sequenced strains

### Validation of the CoCla pipeline

Contig Classifier (CoCla) script validation was performed through reconstruction of the ancient genome of *H. pylori* from the 15 *H. pylori*-enriched samples collected from Ötzi’s stomach. Alignment of the ancient *H. pylori* and the reference strain *H. pylori* 26695 was performed using Mauve software [33]. Furthermore, the BBmap software (sourceforge.net/projects/bbmap/) has been used to detect chimeric contigs through GC content/coverage plot evaluation.

### Metagenome contig selection

After a preliminary assembly with SPAdes [31], each metagenomic sample was analyzed with a customized pipeline aimed at grouping contigs that belong to the same bacterial species. In order to taxonomically classify contigs, open reading frames (ORFs) were predicted with Prodigal [34] and annotated by means of the NCBI database and the aligner RAPSearch2 (cutoff *E* value of 1 × 10^−30^) [35]. Taxonomy information of the best hit obtained for each predicted ORF is used by the CoCla script for contig taxonomy classification based on the most frequently identified microbial species when at least 25% of the ORFs predicted in a contig are attributed to the same species (http://probiogenomics.unipr.it/sw/CoCla.zip). Furthermore, we selected those reads from each shotgun metagenomics dataset that were predicted to belong to a particular bacterial species for which a high-quality and high-coverage assembly was obtained. To achieve this selection, for each metagenomics dataset, the pool of reads was mapped with BBmap (minratio = 0.9 maxindel = 3 minhits = 2 qtrim = r trimq = 10 kfilter = 25 maxsites = 1) (sourceforge.net/projects/bbmap/) on all the assembled contigs as well as publicly available sequences of the selected species. All mapped reads were collected and reassembled with SPAdes in order to obtain high-quality assemblies of a specific species that was shown to be abundantly present in the four metagenomics samples analyzed.

### Sequence annotation

ORFs were predicted using Prodigal [34]. Transfer RNA genes were identified using tRNAscan-SE v1.4 [36], while ribosomal RNA genes were detected using RNAmmer v1.2 [37]. Results of the gene-finder program were combined with data from RAPSearch2 analysis (Reduced Alphabet based Protein similarity Search) [35] of a non-redundant protein database provided by the National Center for Biotechnology Information (NCBI) and Hidden Markov Model profile (HMM) search (http://hmmer.org/) in the manually curated Pfam-A protein family database [38]. The combined results were analyzed through Artemis [39], which was used for a manual-editing effort aimed at verifying and, if necessary, redefining the start of each predicted coding region, to remove or add coding regions, and to discard small ORFs at the edge of the contigs as well as contigs less than 500 bp in length.

### Pan-genome and identification of shared and unique genes

The pan-genome calculation was performed using the PGAP pipeline [40]. The ORF content of all genomes was organized in functional gene clusters using the GF (Gene Family) method involving comparison of each protein to all other proteins using BLAST analysis (cutoff *E* value of 1 × 10^−5^ and 50% identity across at least 50% of both protein sequences), followed by clustering into protein families, named clusters of orthologous genes (COGs), using MCL (graph-theory-based Markov clustering algorithm) [41]. A pan-genome profile was built using an optimized algorithm incorporated in PGAP software, based on a presence/absence matrix that included all identified COGs in the analyzed genomes. Following this, the unique protein families for each genome were classified. Protein families shared between all genomes, named core COGs, were defined by selecting the families that contained at least one single protein member for each genome.

### Phylogenetic and phylogenomic comparisons

The concatenated core genome sequences were aligned using MAFFT [42], and phylogenetic trees were constructed using the neighbor-joining method in Clustal W, version 2.1 [43]. The core genome supertree was built using FigTree (http://tree.bio.ed.ac.uk/software/figtree/). For each genome pair, a value for the average nucleotide identity (ANI) was calculated using the software program JSpecies, version 1.2.1 [44].

### Gene gain/loss through evolution reconstruction

Acquisition and loss of genes through evolution of the bacterial species with at least four available genomes was performed with Count software [45] using Dollo’s parsimony.

### Evaluation of genome sequences and gene distributions

Whole genome sequence alignments for genome coverage analysis were performed at DNA level using LAST (http://last.cbrc.jp/). Prediction of the antibiotic resistance determinants were performed with Rapsearch against a custom database and the Transporter Classification Database (TCDB) (cutoff *E* value of 1 × 10^−30^ and minimum alignment length 50 nucleotides) [46]. The identification of putative virulence genes was carried out by employing the Virulence Factors Database (VFDB) (cutoff *E* value of 1 × 10^−30^ and minimum alignment length 50 nucleotides) [47]. In silico identification of α, β, ε, and ι *C. perfringens* toxins were performed using a custom database based on NCBI RefSeq gene sequences.

### Functional analyses

The prediction of genes that possess structurally related catalytic and carbohydrate-binding modules of enzyme that degrade, modify, or create glycosidic bounds were performed through the CAZy database [48]. A survey of the complete pathways involved in both primary and secondary metabolism has been performed by means of the MetaCyc metabolic pathways database [49]. Each collected gene retrieved from NCBI genomes and the reconstructed ones were taken in account for the screenings using a cutoff *E* value of 1 × 10^−10^ to identify the best hit from each database.

### Taxonomic cataloging of bacteria

Taxonomic classification of reads was obtained using RAPSearch2 software [35] for sequence homology in the NCBI nr database, followed by data processing using MEGAN5 software [50].

## Results and discussion

### Ötzi distal gut microbiome composition

**Fig. 1:**
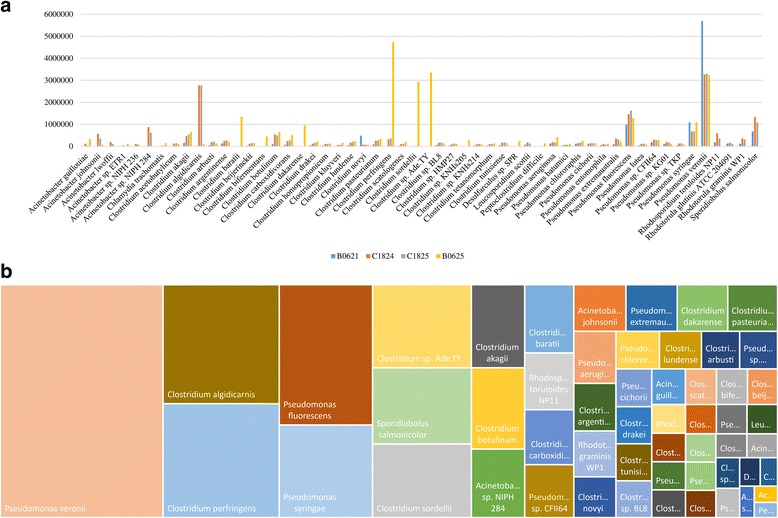
Bacterial abundance in the Ötzi’s gut. Panel **a** displays a bar plot with the abundance of the major species identified in the Tyrolean Iceman gut using CoCla script. The *x axis* represents the identified bacterial species, while the *y* axis represents the number of nucleotides assembled in contigs. Each color reflects a specific sample, i.e., B0625 (*lower part of the large intestine*), C1824 and C1825 (*upper part of the large intestine*), and B0621 (small intestine). Panel **b** shows the visual abundance of the identified species

The substantial amount of available sequence data, i.e., 71 gigabases, from the 12 biopsy samples of the gastrointestinal tract of Ötzi has not yet been fully scrutinized and thus represents an intriguing opportunity for further, in depth functional and genomic studies of the ancient microbiota harbored by the Tyrolean Iceman [21]. Notably, these shotgun data were obtained using protocols specific for processing of ancient DNA and executed in a dedicated facilitate so as to prevent the introduction of any contaminating sequences (see “Methods” section for details). Using the CoCla script, we were able to assembly the four intestinal shotgun metagenomic datasets and to elucidate the overall composition of the reconstructed microbiome down to species level. Notably, a substantial proportion of shotgun sequencing data in the samples was predicted to belong to *Pseudomonas veronii* (13.9%) and *Clostridium algidicarnis* (5.1%) species, followed by *Pseudomonas fluorescens* (4.8%), *Clostridium perfringens* (4%), *Pseudomonas syringae* (3.2%) and *Clostridium* sp. Ade.TY (3%) taxa (Additional file 1: Table S3). Interestingly, samples from the lower part of the large intestine (sample B0625) showed a predominant abundance in *Clostridium* species, while samples from the upper part of the large intestine and the small intestine (C1824, C1825, and B0621) produced sequence data that indicated an abundance of *Pseudomonas* species (Fig. 1). Notably, while clostridia are typical inhabitants of the distal intestine [1], often linked to pathologic conditions [4], *P. fluorescens* is typically found in the soil except for specific conditions such as inflammatory bowel disease [51, 52]. In order to validate the functionality of our customized script, we performed an identity sequence analysis based on the reads retrieved from the Ötzi metagenome samples. The resulting information, following a processing step by MEGAN5 software, revealed an abundance of reads belonging to *C. perfringens* in the lower part of the large intestine, or belonging to *P. fluorescens* in the other three samples from the upper part of the large intestine (Additional file 2: Figure S1). This analysis therefore generated interesting metagenomic data which allowed the genomic reconstruction of the identified prehistoric bacterial species.

In order to evaluate the percentage of the ancient genomes that were covered through targeted genome reconstruction, the obtained contigs were aligned to reference genomes retrieved from the NCBI database. Such analysis showed high coverage (>74.7%) of the *Clostridium* sp. Ade.TY, *C. algidicarnis*, *C. perfringens*, *P. fluorescens*, and *P. veronii* genomes. In contrast, *P. syringae* displayed only a partial coverage (34.1%) of the genome and for this reason was omitted from further analyses (Table 1).

### Validation of ancient bacterial DNA origin and identification of putative microbial contaminants

It has been described that bacterial DNA extracted from ancient samples may be compromised by contamination [53]. In order to verify that the genomes that we reconstructed from Otzi samples are truly of an ancient origin, we performed a sequence damage analysis using the mapDamage software [32]. As shown in Additional file 3: Figure S2, *Clostridium* sp. ADE, *C. algidicarnis* CALG, *C. perfringens* CPER, and *P. veronii* PVER reads display an increased C to T mis-incorporation pattern towards their 5′ end (from 5.7 to 9%), indicative of ancient DNA. In contrast, *P. fluorescens* PFLU shows a substantially lower substitution pattern (3.3%), suggesting that this reconstructed genome originated as a DNA contamination of a modern *P. fluorescens*.

### Validation of the CoCla pipeline for assembly of ancient DNA and screening for chimeric contigs

The script CoCla was evaluated for read selection reliability and assembly performance when it is employed to process ancient DNA. The 15 *H. pylori*-enriched shotgun datasets retrieved from the stomach of Ötzi were used to reconstruct the ancient *H. pylori* genome and its coverage with respect to the modern strain *H. pylori* 26695. The results from this analysis were then compared to those obtained by Maixner et al. [21]. Notably, while Maixner et al. achieved an overall genome coverage that ranged from 84.4 to 92.1%, when aligning reads to the reference genome *H. pylori* 26695 [21], the reconstructed ancient genome of *H. pylori* obtained through CoCla pipeline showed a coverage of 92.4% (Additional file 4: Figure S3). Moreover, BBmap software was used for the screening of chimeric contigs, i.e., assembled sequences encompassing exogenous DNA. The results, reported in Additional file 5: Figure S4, highlight the presence of a single cluster in CG content/coverage plots of all the ancient species assembled, thus indicating the (near) absence of alien DNA.

### Genomic comparisons of the reconstructed bacterial chromosomes with the currently known microbial genomes

Investigation of the general genome features of the reconstructed chromosomes displayed a genome size ranging from 2,713,669 (*Clostridium* sp. CADE) to 6,299,241 (*P. veronii* PVER), corresponding to 2605 and 5805 predicted protein-encoding genes, respectively (Table 1). Furthermore, the average nucleotide identity (ANI) was estimated between each genome belonging to the same species in order to verify the taxonomic classification of the reconstructed ancient genomes and to evaluate their divergence from “modern”’ strains [54]. Two of the assembled genomes exhibit an ANI value higher than 96% when compared with publicly available genome sequences of the same species, which represent recent isolates, e.g., *C. algidicarnis* B3-*C. algidicarnis* CALG (98.38%), and *C. perfringens* SM101-*C. perfringens* CPER (98.46%) pairs. The *P. veronii* PVER genome exhibits an average borderline ANI value of 93.89% with four publicly available *P. veronii* strains (Table 1). In contrast, the reconstructed genomes of *Clostridium* sp. CADE display an ANI value of 90.04% when aligned with the chromosome of *Clostridium* sp. Ade.TY. Thus, the ancient *Clostridium* sp. CADE may be considered a new *Clostridium* taxon, possibly representing the ancestor of the currently existing *Clostridium* sp. Ade.TY.

### Ancient genome evolution through phylogenomic analysis

**Fig. 2:**
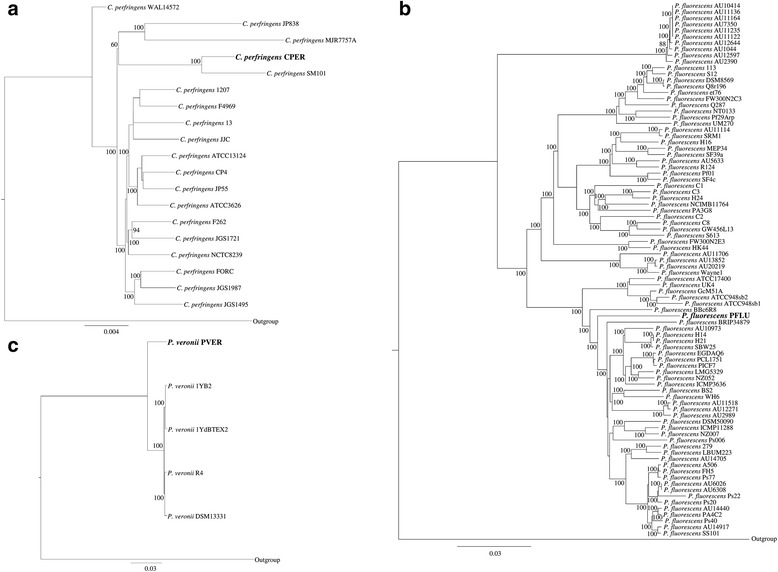
Phylogenetic diversity of the reconstructed ancient genomes. Panel **a** depicts a phylogenetic supertree based on the sequences of identified core proteins shared by the analyzed *C. perfringens* genomes. Panel **b** shows the same supertree based on the analyzed *P. fluorescent* genomes, while panel **c** displays the *P. veronii* supertee

The availability of the reconstructed genomes harbored in Ötzi’s gut allowed us to obtain insights into the genetic makeup of these ancient bacterial strains. The reconstructed ancient genomes were compared to all publicly available genomic sequences belonging to the same species. In order to reduce the bias caused by different analytical genome pipelines, all genomes retrieved from the NCBI database were processed through the same annotation pipeline adopted for the ancient microbial genomes assembled as part of the current study. Notably, as shown in Table 1, core genome analyses highlight a pool of core genes for each identified species ranging from 1737 to 4200 for *C. perfringens* and *P. veronii*, respectively. In order to study the phylogenetic relatedness between the reconstructed ancient genomes and the modern ones, the shared core genome-encoded amino acid sequences were compared. Only the species possessing at least four sequenced genomes in the NCBI database were evaluated. Thus, we excluded from this analysis the genomes of *Clostridium* sp. CADE and *C. algidicarnis* CALG. A concatenated protein sequence based on 1570 of the core CperCOGs (*C. perfringens*-specific clusters of orthologous genes), excluding the duplicated genes that appear to be paralogs, was built for the *C. perfringens* species (Fig. 2). In a similar manner, 3629 PverCOGs (*P. veronii*-specific clusters of orthologous genes) were selected in order to design an analogous supertree for the *P. veronii* species (Fig. 2). Those core COG collections represent the most updated core genome sequences of the *C. perfringens* and *P. veronii* species, and from these, a robust reconstruction of the species phylogeny can be inferred [55, 56]. Investigation of the generated phylogenomic results showed that the *P. veronii* supertree constitutes a single branch, while the *C. perfringens* supertree displayed two major branches (Fig. 2).

Interestingly, the ancient genome of *C. perfringens* CPER shares the same phylogenetic branch with the enterotoxin-producing food poisoning strain SM101, a transformable derivative of *C. perfringens* NCTC 8798 [57]. This discovery, together with the high ANI values observed with *C. perfringens* SM101 (98.46), suggests that *C. perfringens* CPER is a human food poisoning strain related to the well-characterized modern strains. Furthermore, the reconstructed *P. veronii* PVER genome does not share the same branch of the other four sequenced *P. veronii* strains, showing a higher degree of sequence variation within its core genome. These observations suggest that only *C. perfringens* CPER possesses a core genome that can be directly compared with a contemporary genome of the same species, while *P. veronii* PVER appears to have a genomic composition that is specific and different compared to that of modern strains. These data are probably linked to the observation that the ancient strain of *P. veronii* PVER seems to have colonized Ötzi’s gut, while the modern ones have been isolated from natural mineral water springs [58]. Notably, while we cannot completely exclude that the colonization of Ötzi’s large intestine by *P. veronii* PVER may have derived from post-mortem environmental contamination, its abundance and the presence of modern strains in springs may also be due to post-mortem overgrowth of a resident or transient *P. veronii* PVER that had a dietary origin.

### Genome evolution trends in modern vs. ancient genomes

In order to evaluate the presence of horizontal gene transfer events (HGT), all genes were classified by a BLASTp homology search (cutoff *E* value of 1 × 10^−30^) using the NCBI database. Notably, 68.4% of the truly unique genes (TUGs) of *C. perfringens* CPER appear to be unrelated to any genes belonging to members of this genus and 66.7% of these TUGs fail to share homology with any known protein sequence (Additional file 6: Figure S5 and Additional file 1: Table S5). Similarly, it was not possible to identify known homologs in the NCBI database for 47.8% of the TUGs identified in *P. veronii* PVER, suggesting that these TUGs may have been acquired by HGT or have been lost by modern strains.

Focusing on the virulence genes defined in the VF database, it was possible to predict their acquisition and/or loss during the evolution of the analyzed species with at least four available genomes, based on the phylogeny reconstructed by the core gene-based supertrees (Additional file 6: Figure S5). This analysis showed that a large majority (98.1%) of the virulence factors of the *C. perfringens* pan-genome appears to have been acquired early by a species ancestor, thus being shared by all currently known *C. perfringens* strains (Additional file 6: Figure S5). Gene gain/loss reconstruction during evolution of *P. veronii* PVER suggests that this strain possesses 8% less virulence genes compared to the average of modern strains of the same species. This suggests that over the last 3500 years acquisition of virulence genes factors was one of the main evolutionary drivers for this taxon (Additional file 6: Figure S5).

### Ancient unique genomic regions of Ötzi’s microbiome

The Pan-genome predictions allowed the identification of TUGs representing Unique Genomic Regions (UGRs) belonging to the reconstructed genomes of the gut microbiota members harbored by the Tyrolean Iceman (Table 1 and Additional file 1: Table S5). The comparisons of *Clostridium* sp. CADE and *C. algidicarnis* CALG with the publicly available genomic sequences of modern relatives revealed 215 and 216 TUGs, respectively, while *C. perfringens* CPER exhibits a higher number of TUGs among the ancient *Clostridium* species (i.e., 493) (Table 1). Moreover, *P. veronii* PVER appears to contain a higher number of unique genes, i.e., 615 TUGs, which is probably a reflection of its larger genome size.

**Fig. 3:**
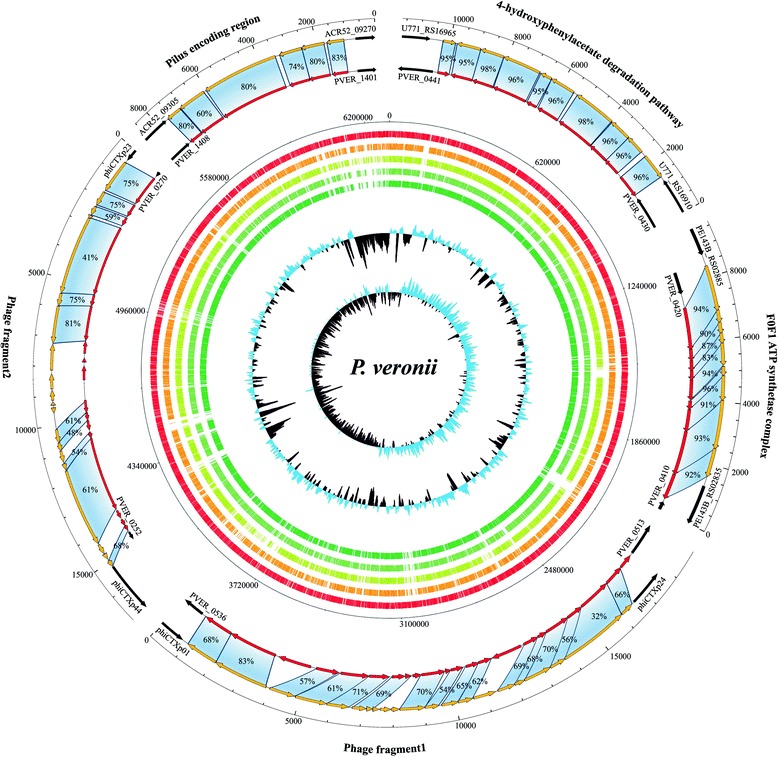
Comparative genomic analysis of *P. veronii* PVER with other fully sequenced *P. veronii* strains. Circular genome atlas of *P. veronii* PVER (*red circle*) with mapped orthologues (defined as reciprocal best BLASTp hits with more than 50% identity over at least 50% of both protein lengths) in four publicly available *P. veronii* genomes (*orange through green circle*). Internal circles illustrate *P. veronii* PVER GC% deviation and GC skew (G − C/G + C), while the external maps exhibit the sequence identity between the unique loci of *P. veronii* PVER compared to other bacteria retrieved from the database. Each *arrow* indicates an ORF, whereas the length of the arrow is proportional to the length of the predicted ORF. *Red arrows* correspond to the *P. veronii* PVER genes, while *orange arrows* display orthologous genes

Genome comparisons between the chromosome sequence of *Clostridium* sp. Ade.TY and those of modern relatives revealed the absence of a major region (Fig. 3 and Additional file 7: Figure S6), which contains a gene cluster predicted to encode a β-d-glucuronide and d-glucuronate degradation super-pathway and a smaller region with a complete phosphotransferase system (PTS), highlighting additional capability in the uptake of carbohydrates and energy production. In contrast, *C. algidicarnis* CALG possesses (compared to the genome of *C. algidicarnis* B3) many unique, yet small genomic regions scattered with genes coding for hypothetical proteins. Notably, one of these unique *C. algidicarnis* CALG regions encompasses a complete type III restriction modification system (RM system), which is absent in the modern strain. The *C. perfringens* CPER atlas analysis showed that the genome of *C. perfringens* str. 13 possesses a very simplified genome, while the other 16 publicly available strains analyzed showed the widespread presence of unique genomic regions that all appear to be present in the reference genome of *C. perfringens* CPER. These data suggest that *C. perfringens* CPER probably represents an ancestral genome with higher genetic complexity than the modern *C. perfringens* strains, which seems to have undergone an innovation phase that was aimed at increasing genome complexity without following a reductive phase to achieve genome simplification by gene loss.

Interestingly, the genome of *P. veronii* PVER, when aligned with the four chromosome sequences of contemporary *P. veronii* strains, shows many unique genomic regions, rendering this genome one of the most interesting ancient bacterial genomes in terms of identified unique genes. Remarkably, the most characteristic unique loci were shown to include a prophage, a F0F1 ATP synthetase complex, a 4-hydroxyphenylacetate degradation pathway and a pilus-encoding region (Fig. 3). Notably, a comparison of the *P. veronii* PVER prophage’s unique gene content reveals extensive homology with phage CTX isolated from *Pseudomonas aeruginosa* [59]. More than half of the total ORFeome of the identified prophage (29) displays a marked homology (from 43.1 to 83.4%) to φCTX genes (Fig. 3). The unique pilus-encoding genes show high sequence identity (from 59.5 to 83.4%) to homologs located on the genome of *Pseudomonas* sp. KG01, isolated from the Antarctic (Fig. 3). Interestingly, the publicly available *P. veronii* strains were isolated from soil and water samples [58]; thus, we can assume that the ancient *P. veronii* PVER may have possessed peculiar systems that assisted this strain in human gut colonization. Accordingly, the modern *P. veronii* strains may have lost the pilus-encoding capacity, perhaps together with its capability to colonize the human gut. Furthermore, the unique F0F1 complex identified in *P. veronii* PVER shares high sequence identity to the same complex of *Pseudomonas extremaustralis* strain 14–3 (from 84.3 to 95.8%) (Fig. 3), a bacterium isolated from a temporary pond in Antarctica. Interestingly, the phylogenetic relatedness between *P. extremaustralis* strain 14–3 and other *P. veronii* strains had been described previously [60].

### Toxin-encoding genes and antibiotic resistance in the ancient Ötzi strains

Since members of the *Clostridium* genus produce a wide range of toxins and secreted virulence factors [61], the toxin and virulence factor-encoding genes are intensively exploited as a key typing system for *C. perfringens*. This species is one of the most prolific producers of toxins [62], with five biotypes (A–E) delineated on the basis of the differential production of α, β, ε, and ι toxins [55]. Using a custom database composed of toxin sequences of well-known *C. perfringens* strains, we were able to predict the toxins that may be synthetized by *C. perfringens* CPER. Specifically, the prehistoric *C. perfringens* CPER was predicted to encode the α-toxin (phospholipase C) associated with the biotype A of *C. perfringens* species (99.2% sequence identity compared to that encoded by the NRRL B-23841 strain) (Table 2). Interestingly, in humans, biotype A is typically associated with food poisoning [55]. Furthermore, thanks to the virulence factor and NCBI databases, we were able to identify other *C. perfringens* toxins in the reconstructed ancient genome that are currently not used as biotype delineators, such as β2-toxin (CPB2), θ-toxin (perfringolysin O), μ-toxin (hyaluronidase), κ-toxin (collagenase), and several enterotoxins (CPE) (Table 2). Notably, β2-toxin is associated with enteritis in animals [63], while θ-toxin can damage blood vessels, resulting in leukostasis, thrombosis, and tissue hypoxia. More interestingly, the high number of enterotoxins indicates a high probability that *C. perfringens* CPER was able to cause (symptoms of) food poisoning, as also suggested by the fact that modern CPE-producing type A strains are major human gut pathogens [64]. However, the reconstructed genome of *C. perfringens* CPER strain does not include those toxins that can ultimately lead to death in humans (β and ε toxins), indicating that this particular pathogenic characteristic may have been introduced in the modern strains in order to increase the ecological fitness of the *C. perfringens* species.

**Table 2:** *Clostridium perfringens* toxins

CPER ORFs	VFDB subject	Identity	Aln-len	Mismatch	Gap openings	Q.start	Q.end	S.start	S.end	log (*e* value)	Bit score	Predicted annotation
CPER_0621	VFG002284 (gi:18309535) (nanJ) exo-alpha-sialidase [sialidase (VF0391)] [*Clostridium perfringens* str. 13]	94.4	1173	66	0	1	1173	1	1173	−10000	2225.7	Exo-alpha-sialidase
CPER_0726	VFG002274 (gi:18309018) (plc) phospholipase C [alpha-toxin (CpPLC) (VF0378)] [*Clostridium perfringens* str. 13]	98.2	398	7	0	1	398	1	398	−240.2	826.2	Phospholipasae C
CPER_0764	VFG002279 (gi:18310216) (nagJ) hyaluronidase [mu-toxin (VF0389)] [*Clostridium perfringens* str. 13]	98.1	879	17	0	1	879	1	879	−10000	1726.1	Hyaluronidase
CPER_1090	VFG002276 (gi:18309155) (colA) collagenase [kappa-toxin (VF0388)] [*Clostridium perfringens* str. 13]	98.3	1043	18	0	1	1043	62	1104	−10000	2096.2	Peptidase M9
CPER_1851	VFG002278 (gi:18309863) (nagI) hyaluronidase [mu-toxin (VF0389)] [*Clostridium perfringens* str. 13]	98.6	1297	18	0	1	1297	1	1297	−10000	2558.1	Hyaluronidase
CPER_2203	VFG002282 (gi:18309828) (cloSI) alpha-clostripain [alpha-clostripain (VF0390)] [*Clostridium perfringens str*. 13]	97.3	522	14	0	1	522	3	524	−304.5	1040	Alpha-clostripain precursor
CPER_2247	VFG002280 (gi:18310261) (nagK) hyaluronidase [mu-toxin (VF0389)] [*Clostridium perfringens* str. 13]	99.3	1157	8	0	1	1157	1	1157	−10000	2315.8	Hyaluronidase
CPER_2490	VFG002285 (gi:110800384) (nanH) sialidase [sialidase (VF0391)] [*Clostridium perfringens* ATCC 13124]	95.9	294	12	0	1	294	89	382	−168.5	587.4	Exo-alpha-sialidase
CPER_2569	VFG002277 (gi:18309173) (nagH) hyaluronidase [mu-toxin (VF0389)] [*Clostridium perfringens* str. 13]	98	1058	21	0	1	1058	21	1078	−10000	2120.1	Hyaluronoglucosaminidase
CPER_2658	VFG002283 (gi:18309707) (nanI) exo-alpha-sialidase [sialidase (VF0391)] [*Clostridium perfringens* str. 13]	99.7	694	2	0	1	694	1	694	−10000	1404.4	Exo-alpha-sialidase
CPER_2774	VFG002281 (gi:18310505) (nagL) hyaluronidase [mu-toxin (VF0389)] [*Clostridium perfringens* str. 13]	97.9	900	19	0	9	908	1	900	−10000	1787.3	Hyaluronidase
CPER_2934	VFG002275 (gi:18309145) (pfoA) perfringolysin O [theta-toxin/PFO (VF0382)] [*Clostridium perfringens* str. 13]	99.8	500	1	0	1	500	1	500	−289.6	990.7	Perfringolysin O

Moreover, the prediction of toxin-antitoxin systems (TA) was performed for the complete reconstructed genomes, in order to explore the capabilities of these strains to control cell growth and bacterial persistence. The generated information revealed the presence of a small number of complete TA systems in the *Clostridium* genomes, ranging from one in *Clostridium* sp. CADE to three in *C. perfringens* CPER, and an extraordinary richness of 11 such systems in the *P. veronii* PVER chromosome (Table 3). Recently, a high abundance in TA systems has been correlated with pathogenicity of epidemic bacteria such as *Mycobacterium tuberculosis* [65], while TA systems are more abundant in free-living microorganisms rather than host-associated species [66]. Thus, *P. veronii* PVER does not seem to possess a distinguishing TA system-associated profile of host-associated commensals, but, rather, a profile that is reminiscent of highly pathogenic bacteria adapted to survive and persist in their environment.

**Table 3:** Predicted toxin-antitoxin systems

Ancient ORFs	TADB code	TADB gene	Subject	Identity	Aln-len	Mismatch	Gap openings	Q.start	Q.end	S.start	S.end	Log (*e* value)	Bit score	Predicted annotation
CADE_0165	TADB|8353403	CLL_A3362	*Clostridium botulinum* B str. Eklund 17B	96.6	116	4	0	1	116	1	116	-57.5	216.9	mRNA interferase PemK
CADE_0166	–	–	–	–	–	–	–	–	–	–	–	–	–	Hypothetical protein
CALG_0512	TADB|8436183	CLH_2005	*Clostridium botulinum* E3 str. Alaska E43	76.7	146	34	0	1	146	1	146	−64.3	240	Hypothetical protein
CALG_0513	TADB|8436181	CLH_2004	*Clostridium botulinum* E3 str. Alaska E43	63.8	127	46	0	4	130	6	132	−45.3	176.4	Hypothetical protein
CALG_1265	TADB|375739	CAC0494	*Clostridium acetobutylicum* ATCC 824	90.4	114	11	0	3	116	2	115	−54.3	206.5	mRNA interferase PemK
CALG_1266	–	–	–	–	–	–	–	–	–	–	–	–	–	CopG family transcriptional regulator
CPER_1456	TADB|4710268	CPF_0812	*Clostridium perfringens* ATCC 13124	95.8	71	3	0	1	71	1	71	−35.6	144.4	Hypothetical protein
CPER_1457	TADB|607725	CPE0814	*Clostridium perfringens* str. 13	97.5	162	4	0	1	162	1	162	−87.9	318.2	GNAT family acetyltransferase
CPER_2012	TADB|4710831	CPF_1033	*Clostridium perfringens* ATCC 13124	99.4	168	1	0	1	168	1	168	−95.5	343.6	Hypothetical protein
CPER_2013	TADB|4710829	CPF_1032	*Clostridium perfringens* ATCC 13124	100	69	0	0	1	69	1	69	−34	139	DNA-binding protein
CPER_2208	TADB|4721113	CPR_0896	*Clostridium perfringens* SM101	97.2	143	4	0	1	143	1	143	−77.7	284.3	Hypothetical protein
CPER_3173	TADB|4710797	CPF_1020	*Clostridium perfringens* ATCC 13124	97.9	140	3	0	7	146	1	140	−75.2	275.8	Hypothetical protein
CPER_3174	TADB|4710799	CPF_1021	*Clostridium perfringens* ATCC 13124	95.6	180	8	0	1	180	1	180	−93.5	337	XRE family transcriptional regulator
PFLU_1561	TADB|9079447	PFLU2030	*Pseudomonas fluorescens* SBW25	98.4	123	2	0	1	123	1	123	−64.8	241.1	Hypothetical protein
PFLU_1562	TADB|9079443	PFLU2029	*Pseudomonas fluorescens* SBW25	88.5	113	12	1	1	112	1	113	−50.8	194.9	Cro/Cl family transcriptional regulator
PFLU_2731	–	–	–	–	–	–	–	–	–	–	–	–	–	Hexulose-6-phosphate isomerase
PFLU_2732	TADB|348443	hicB-2	*Pseudomonas putida* KT2440	71.3	108	31	0	5	112	2	109	−42.4	166.8	Antitoxin HicB
PFLU_4077	–	–	–	–	–	–	–	–	–	–	–	–	–	Protein RnfH
PFLU_4078	–	–	–	–	–	–	–	–	–	–	–	–	–	Ribosome association toxin RatA
PVER_0551	TADB|12012688	BCAS0580	*Burkholderia cenocepacia* J2315	55.8	129	56	1	1	128	1	129	−35.9	145.2	Twitching motility protein PilT
PVER_0552	–	–	–	–	–	–	–	–	–	–	–	–	–	AbrB family transcriptional regulator
PVER_1351	TADB|9070051	PFLU0443	*Pseudomonas fluorescens* SBW25	69.9	133	40	0	1	133	1	133	−50.8	194.9	Transcriptional regulator
PVER_1352	TADB|9070048	PFLU0442	*Pseudomonas fluorescens* SBW25	64.2	95	34	0	1	95	1	95	−33.8	138.3	Hypothetical protein
PVER_1942	–	–	–	–	–	–	–	–	–	–	–	–	–	Motility quorum-sensing regulator MqsR
PVER_1943	TADB|3146921	PFL_1052	*Pseudomonas fluorescens* Pf-5	58.1	136	55	1	1	136	1	134	−38.8	155.2	Transcriptional regulator
PVER_2079	TADB|9097595	PFLU5131	*Pseudomonas fluorescens* SBW25	94	84	5	0	1	84	19	102	−39.7	157.9	Prevent-host-death protein
PVER_2080	TADB|9097599	PFLU5132	*Pseudomonas fluorescens* SBW25	95.2	84	4	0	1	84	1	84	−44.9	175.3	Toxin YoeB
PVER_2187	–	–	–	–	–	–	–	–	–	–	–	–	–	Addiction module toxin RelE
PVER_2188	TADB|9082037	PFLU2450	*Pseudomonas fluorescens* SBW25	92	100	8	0	1	100	1	100	−47.6	184.1	Transcriptional regulator
PVER_2192	TADB|9082090	PFLU2458	*Pseudomonas fluorescens* SBW25	92.8	125	9	0	1	125	1	125	−62.2	232.6	Twitching motility protein PilT
PVER_2193	TADB|9082094	PFLU2459	*Pseudomonas fluorescens* SBW25	76.9	117	27	0	1	117	1	117	−47.4	183.3	Prevent-host-death protein
PVER_2252	TADB|348443	hicB-2	*Pseudomonas putida* KT2440	71.3	108	31	0	5	112	2	109	−42.4	166.8	Antitoxin HicB
PVER_2253	–	–	–	–	–	–	–	–	–	–	–	–	–	Hexulose-6-phosphate isomerase
PVER_2785	TADB|9079443	PFLU2029	*Pseudomonas fluorescens* SBW25	88.5	113	12	1	1	112	1	113	−50.8	194.9	Cro/Cl family transcriptional regulator
PVER_2786	TADB|9079447	PFLU2030	*Pseudomonas fluorescens* SBW25	98.4	123	2	0	1	123	1	123	−64.8	241.1	Hypothetical protein
PVER_3149	TADB|3145803	PFL_0652	*Pseudomonas fluorescens* Pf-5	78.7	94	20	0	1	94	1	94	−37.3	149.8	Addiction module antitoxin
PVER_3150	TADB|3145805	PFL_0653	*Pseudomonas fluorescens* Pf-5	59.6	99	40	0	1	99	12	110	−30.3	126.7	Addiction module protein
PVER_3532	TADB|9094658	PFLU4639	*Pseudomonas fluorescens* SBW25	91.5	117	10	0	1	117	2	118	−57	215.3	Transcriptional regulator
PVER_3533	TADB|9094655	PFLU4638	*Pseudomonas fluorescens* SBW25	88	100	12	0	1	100	1	100	−46.4	180.3	Toxin RelE
PVER_4905	–	–	–	–	–	–	–	–	–	–	–	–	–	Protein RnfH
PVER_4906	–	–	–	–	–	–	–	–	–	–	–	–	–	Ribosome association toxin RatA

Additionally, an antibiotic resistance (AR) gene profiling was conducted on the identified genes of the ancient Ötzi’s strains and the related publicly available genomes. The screening highlighted abundance in putative beta-lactamase and glycopeptide resistance proteins as well as various transporters belonging to the ATP-binding cassette (ABC) uptake porters and major facilitator superfamily (MFS). Interestingly, modern strains appear to possess a larger arsenal of AR-encoding genes. In particular, modern *P. veronii* and *Clostridium* genomes show 24% and up to 17% more AR genes compared to their ancient relatives *P. veronii* PVER and *C. algidicarnis* CALG, respectively. Such observations reveal that the genomes of modern strains have developed or horizontally acquired a larger AR-encoding gene arsenal, perhaps in order to counteract the introduction of antibiotics in the modern era.

### Functional profiling of prehistoric microbial genomes

A functional profiling as well as an enzymatic gene classification was performed to evaluate the metabolic pathways encoded by the reconstructed prehistoric genomes. This in silico metabolic profiling showed that the ancient strains encode various complete pathways that appear to be absent in modern strains, e.g., the “preQ0 biosynthesis” of *Clostridium* sp. CADE, as well as absence of complete pathways identified in the modern strains, e.g., the “myo-, chiro-, and scillo-inositol degradation” in *C. algidicarnis* CALG and the “pyochelin biosynthesis” in *P. veronii* PVER (Table 4). Interestingly, *Clostridium* sp. CADE lacks the “sucrose degradation I (sucrose phosphotransferase)” pathway, as well as alternative sucrose degradation pathways, indicating that the ancient strain was not able to use sucrose in order to produce β-d-glucose 6-phosphate and β-d-fructofuranose 6-phosphate as glycolytic substrates. In contrast, as shown from the unique loci analysis, a complete pathway for the “β-d-glucuronide and d-glucuronate degradation” was detected, highlighting the capability to produce d-fructuronate, which may then be further degraded into d-glyceraldehyde 3-phosphate and pyruvate (Table 4). Another intriguing finding with respect to the differences in metabolic capabilities between prehistoric vs. contemporary strains is represented by the ability to degrade 4-hydroxyphenylacetate. *P. veronii* PVER, as discussed before, shows a complete 4-hydroxyphenylacetate degradation pathway already identified in other strains belonging to the Proteobacteria phylum, highlighting the capability to produce succinate and pyruvate from alternative carbon sources (Table 4).

**Table 4:** Ancient metabolic pathways

Species	Pathways	Status	Enzymes	Genes	Starting molecule	Final product
*Clostridium* sp. CADE	PreQ0 biosynthesis	Complete	GTP cyclohydrolase I	*folE*	guanosine 5′-triphosphate (GTP)	7-cyano-7-deazaguanine (preQ0)
			6-carboxy-5,6,7,8-tetrahydropterin synthase	*queD*		
			7-carboxy-7-deazaguanine synthase	*queE*		
			7-cyano-7-deazaguanine synthase	*queC*		
	β-d-glucuronide and d-glucuronate degradation	Complete	β-d-glucuronidase	*uidA*	β-d-glucuronoside	d-fructuronate
			d-glucuronate isomerase	*uxaC*		
			d-mannonate oxidoreductase	*uxuB*		
			d-mannonate dehydratase	*uxuA*		
			2-keto-3-deoxygluconokinase	*kdgK*		
			2-keto-3-deoxygluconate 6-phosphate aldolase	*eda*		
	Flavin biosynthesis I	Partial	bifunctional riboflavin kinase/FMN adenylyltransferase	*ribF*	Guanosine 5′-triphosphate (GTP)	Flavin adenine dinucleotide (FAD)
			5-amino-6-(5-phospho-d-ribitylamino) uracil phosphatase	*ybjI*		
			3,4-dihydroxy-2-butanone 4-phosphate synthase	*ribB*		
	Biotin biosynthesis from 8-amino-7-oxononanoate I	Partial	Biotin synthase	*bioB*	8-amino-7-oxononanoate (KAPA)	Vitamin H (biotin)
	Biotin biosynthesis from 8-amino-7-oxononanoate II	Partial	Biotin synthase	*bioB*	8-amino-7-oxononanoate (KAPA)	Vitamin H (biotin)
	Sucrose degradation I (sucrose phosphotransferase)	Absent	–	–	Sucrose	β-d-fructofuranose 6-phosphate (F6P)
*C. algidicarnis* CALG	Myo-, chiro- and scillo-inositol degradation	Partial	(methyl) malonate-semialdehyde dehydrogenase gene	*mmsA*	Inositol	Glycerone phosphate (DHAP) + acetyl-CoA
	Myo-inositol degradation	Partial	(methyl) malonate-semialdehyde dehydrogenase gene	*mmsA*	Inositol	Glycerone phosphate (DHAP) + acetyl-CoA
*P. fluorescens* PFLU	KDO transfer to lipid IVA I	Absent	–	–	CMP-3-deoxy-β-d-manno-octulosonate	α-Kdo-(2- > 4)-α-Kdo-(2- > 6)-lipid IVA
	KDO transfer to lipid IVA II	Absent	–	–	CMP-3-deoxy-β-d-manno-octulosonate	4-O-phospho-α-Kdo-(2 → 6)-lipid IVA
*P. veronii* PVER	Pyochelin biosynthesis	Absent	–	–	l-cysteine	Pyochelin
	4-hydroxyphenylacetate degradation	Complete	4-coumarate 3-monooxygenase	*hpaB*	4-hydroxyphenylacetate	Succinate

**Fig. 4:**
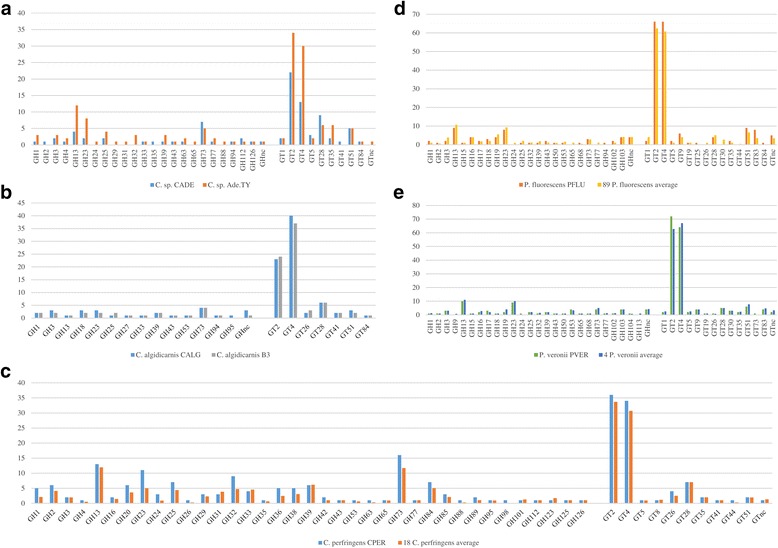
Carbohydrate-active enzymes of the ancient genomes. Panel **a** exhibits a bar plot with the abundance in Glycosyl hydrolase (GH) or Glycosyltransferase (GT) families encoded by the genomes of *Clostridium* sp. CADE and *Clostridium* sp. Ade.TY. Panel **b** shows a similar bar plot for *C. algidicarnis* CALG and *C. algidicarnis* B3, while panels **c**, **d**, and **e** display the GH and GT average between the publicly available genomes of *C. perfringens*, *P. fluorescens* and *P. veronii*, respectively

Investigation of the Carbohydrate Active Enzymes using the CAZY database [48] highlighted intriguing differences between modern and ancient genomes, which may explain a specific adaptation of the modern strains to novel ecological niches. It was noted that the genome of *C.* sp. CADE possesses a lower abundance in Glycosyl Hydrolases (GHs) and Glycosyl Transferases (GTs) compared to the *Clostridium* sp. Ade.TY strain, exhibiting a reduction in genes that encode such enzymes by 46.6 and 33.3%, respectively (Fig. 4). This smaller GH arsenal is in particular due to reduced numbers of members of GH13 and GH23 families, which are predicted to be involved in the hydrolysis of substrates containing α-glucoside linkages such as maltose. In contrast, the genome of *C. perfringens* CPER shows a higher abundance in GHs (28.6%) and GTs (8%) compared to the average of the 18 sequenced modern strains (Fig. 4). A substantial proportion of these GHs belong to the GH23 and GH32 families, highlighting an improved capability for the hydrolysis of fructose-containing sugars compared to the other 18 *C. perfringens* strains. In contrast, the predicted GH and GT repertoire of the *P. veronii* PVER genome, when compared to that of modern strains, revealed a similar composition in its carbohydrate-active enzyme profile (Fig. 4).

## Conclusions

Next generation sequencing (NGS) approaches are a valuable resource allowing the exploration of the composition and functionalities of the human gut microbiota in the soft tissue of human mummies. In this study, we performed an in depth metagenomic analysis of four biopsy samples recently retrieved from the small and large intestines of the Tyrolean Iceman [21], resulting in the reconstruction and characterization of ancient microbial genomes that were predominant in the Tyrolean Iceman’s distal gut at death. The reconstructed genomes of *C. perfringens*, *Clostridium* sp. Ade.TY, *C. algidicarnis*, and *P. veronii* allowed the identification of evolutionary development of these taxa. The reconstructed genome of *Clostridium* sp. CADE displayed high genomic variability with the chromosome of the related *Clostridium* sp. Ade.TY; thus, it may be considered as a novel *Clostridium* species that is capable of degrading glucoronate, yet is unable to metabolize sucrose. Interestingly, analyses performed on the ancient genome *C. perfringens* CPER highlight a similar genome structure and a phylogenetic relatedness with the enterotoxin-producing food poisoning strain *C. perfringens* SM101. Furthermore, the identification of genes that encode the α-toxin (phospholipase C) and various other enterotoxins suggest that *C. perfringens* CPER was a human food poisoning strain associated with the biotype A of toxigenic *C. perfringens* species. Despite the fact that modern *P. veronii* strains have been isolated from water springs, the ancient strain PVER seems to possess the ability to colonize the human gut (pre- or post-mortem). Moreover, *P. veronii* PVER is related to *Pseudomonas* strains isolated from the Antarctic, thus supporting its ancient origin. Notably, gene gain/loss prediction showed that one of the main forces driving the evolution of *P. veronii* was the development and/or acquisition of novel virulence factors. While modern/ancient average ratio of the predicted antibiotic resistances suggests that the modern strains have been subjected to substantial selective pressure, possibly due to the extensive use of antibiotics in the modern era.

## Additional files

Additional file 1:
**Tables S1, S2, S3, S4, and S5.**

Additional file 2: Figure S1. Bacterial abundance in Ötzi’s gut. Panel a displays a bar plot with the abundance of the major species identified in the Tyrolean Iceman gut using MEGAN5 software. The x axis represents the identified bacterial species, while the y axis represents the number of reads. Each color reflects a specific sample, i.e., B0625 (lower part of the large intestine), C1824 and C1825 (upper part of the large intestine), and B0621 (small intestine). Panel b visually displays the observed abundance of the identified species.
Additional file 3: Figure S2. Cytosine to thymine substitution frequency at the 5′ end of the sequenced reads. The plot displays the cytosine deamination pattern of the *Clostridium* sp. CADE, *C. algidicarnis* CALG, *C. perfringens* CPER, *P. fluorescens* PFLU, and *P. veronii* PVER selected reads from the Ötzi’s metagenomic samples. The y axis reports the C to T substitution frequency, while the x axis indicates the distance from the 5′ end of the sequence reads.
Additional file 4: Figure S3. Mauve alignment of reconstructed ancient *H. pylori* to the reference *H. pylori* 26695. On top the reference *H. pylori* 26695 genome is depicted, while the reordered contigs of the reconstructed ancient *H. pylori* from the Ötzi’s metagenomics samples is indicated on the bottom part of the figure. Each colored block corresponds to a conserved genomic region between the two genome sequences, while the red line discerns the ancient *H. pylori* contigs.
Additional file 5: Figure S4. CG content/coverage plot of ancient reconstructed genome contigs. Panel a displays the CG content distribution of the *Clostridium* sp. CADE contigs; similarly, panels b to d display this for *C. algidicarnis* CALG, *C. perfringens* CPER, and *P. veronii* PVER contigs, respectively.
Additional file 6: Figure S5. Evolutionary gain and loss analysis based on predicted virulence factors within *C. perfringens*, *P. fluorescens*, and *P. veronii* species. Panel a displays the core gene-based supertree of *C. perfringens* strains, where each node reports the number of predicted virulence factor COGs identified for each strain. Furthermore, gains and losses are indicated by green and orange bars on the edge leading to each node. Panels b and c show the same analysis conducted on *P. fluorescens* and *P. veronii* species, respectively.
Additional file 7: Figure S6. Comparative genomic analysis of *Clostridium* sp. CADE, *C. algidicarnis* CALG, and *C. perfringens* CPER with other fully sequenced strains. Panel a displays the circular genome atlas of *Clostridium* sp. CADE (red circle) with mapped orthologues (defined as reciprocal best BLASTp hits with more than 50% identity over at least 50% of both protein lengths) in public available *Clostridium* sp. Ade.TY genome (orange circle). Internal circles illustrate GC% deviation and GC skew (G − C/G + C). Panel b and c shows the same circular genome atlas of *C. algidicarnis* CALG and *C. perfringens* CPER, respectively.

## Abbreviations

ABC: ATP-binding cassette
ANI: Average nucleotide identity
AR: Antibiotic resistance
COG: Clusters of orthologous genes
CPE: *Clostridium perfringens* enterotoxins
GF: Gene family
GH: Glycosyl hydrolases
GT: Glycosyltransferases
HGT: Horizontal gene transfer
HMM: Hidden Markov model
MFS: Major facilitator superfamily
NCBI: National Center for Biotechnology Information
NGS: Next-generation sequencing
ORF: Open reading frames
PTS: Phosphotransferase system
TA: Toxin-antitoxin systems
TUG: Truly unique genes
UGR: Unique genomic regions
