# Erratum to: Ancient bacteria of the Ötzi’s microbiome: a genomic tale from the Copper Age

**DOI:** 10.1186/s40168-017-0243-0

**Published:** 2017-02-17

**Authors:** Gabriele Andrea Lugli, Christian Milani, Leonardo Mancabelli, Francesca Turroni, Chiara Ferrario, Sabrina Duranti, Douwe van Sinderen, Marco Ventura

**Affiliations:** 1 Laboratory of Probiogenomics, Department of Life Sciences, University of Parma, Parco Area delle Scienze 11a, 43124 Parma, Italy; 2 APC Microbiome Institute and School of Microbiology, National University of Ireland, Cork, Ireland

## Erratum

Upon publication of the original article [1], it was brought to our attention that there was a conflict between this publication, in the paragraph of the M&M section entitled “Ancient DNA extraction and Illumina libraries preparation”, and the article entitled “The 5300-year-old Helicobacter pylori genome of the Iceman” published in Science in 2016 [2], from where the metagenomic datasets that were analysed in this publication were sequenced. This paragraph merely describes the procedures as used by Maixner et al. for the extraction and processing of the DNA samples (that then generated the microbiome datasets analysed in the study). The authors of both papers wish to clarify that this published study was not carried out in the framework of Dr Maixner’s project.

As such, please see below the modified text of this paragraph, which provides a brief description of the procedures used:

### Ancient DNA extraction and Illumina libraries preparation

The *in silico* analyses performed in this study were carried out using the microbiome data of the Tyrolean Iceman previously sequenced and released by Maixner et al., [21]. For details to the DNA extraction procedures, library preparation and sequencing please refer to the Supplementary Material of this previous study [21]. In brief, DNA extraction using 250 mg of biological samples have been performed with all the stringent laboratory procedures needed for investigations of ancient DNA in the ancient DNA laboratory of the EURAC - Institute for Mummies and the Iceman, Bolzano, Italy [26-27]. Library preparation and Illumina sequencing were performed at the Institute of Clinical Molecular Biology, Kiel University [28, 29].
