# Supplementary figures and images for: Ancient bacteria of the Ötzi’s microbiome: a genomic tale from the Copper Age
Source: Microbiome. 2017 Jan 17;5:5. doi: 10.1186/s40168-016-0221-y (PMC5240250; doi:10.1186/s40168-016-0221-y)

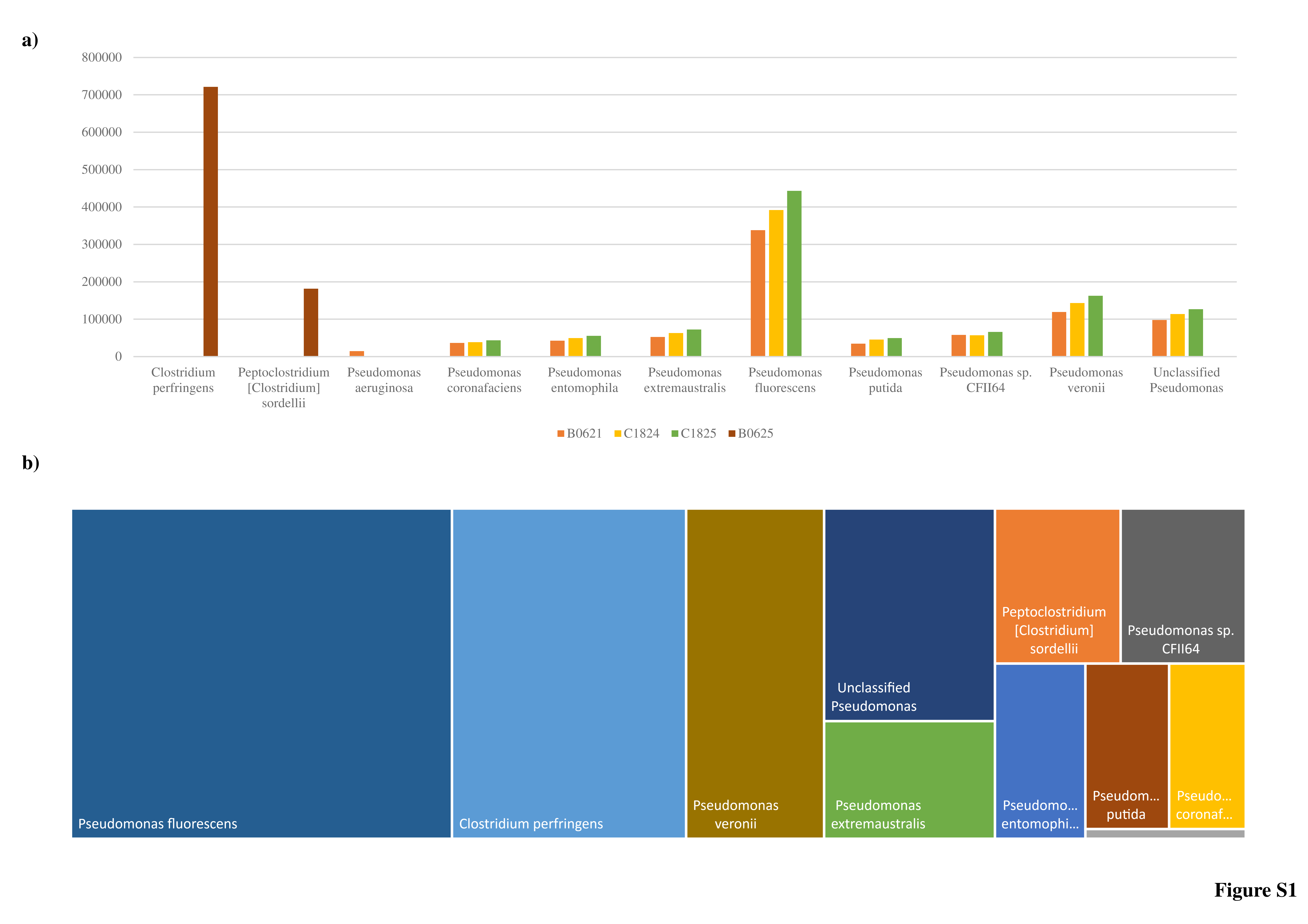

Supplement: Additional file 2: Figure S1. — Bacterial abundance in Ötzi’s gut. Panel a displays a bar plot with the abundance of the major species identified in the Tyrolean Iceman gut using MEGAN5 software. The x axis represents the identified bacterial species, while the y axis represents the number of reads. Each color reflects a specific sample, i.e., B0625 (lower part of the large intestine), C1824 and C1825 (upper part of the large intestine), and B0621 (small intestine). Panel b visually displays the observed abundance of the identified species. [file 40168_2016_221_MOESM2_ESM.tif]

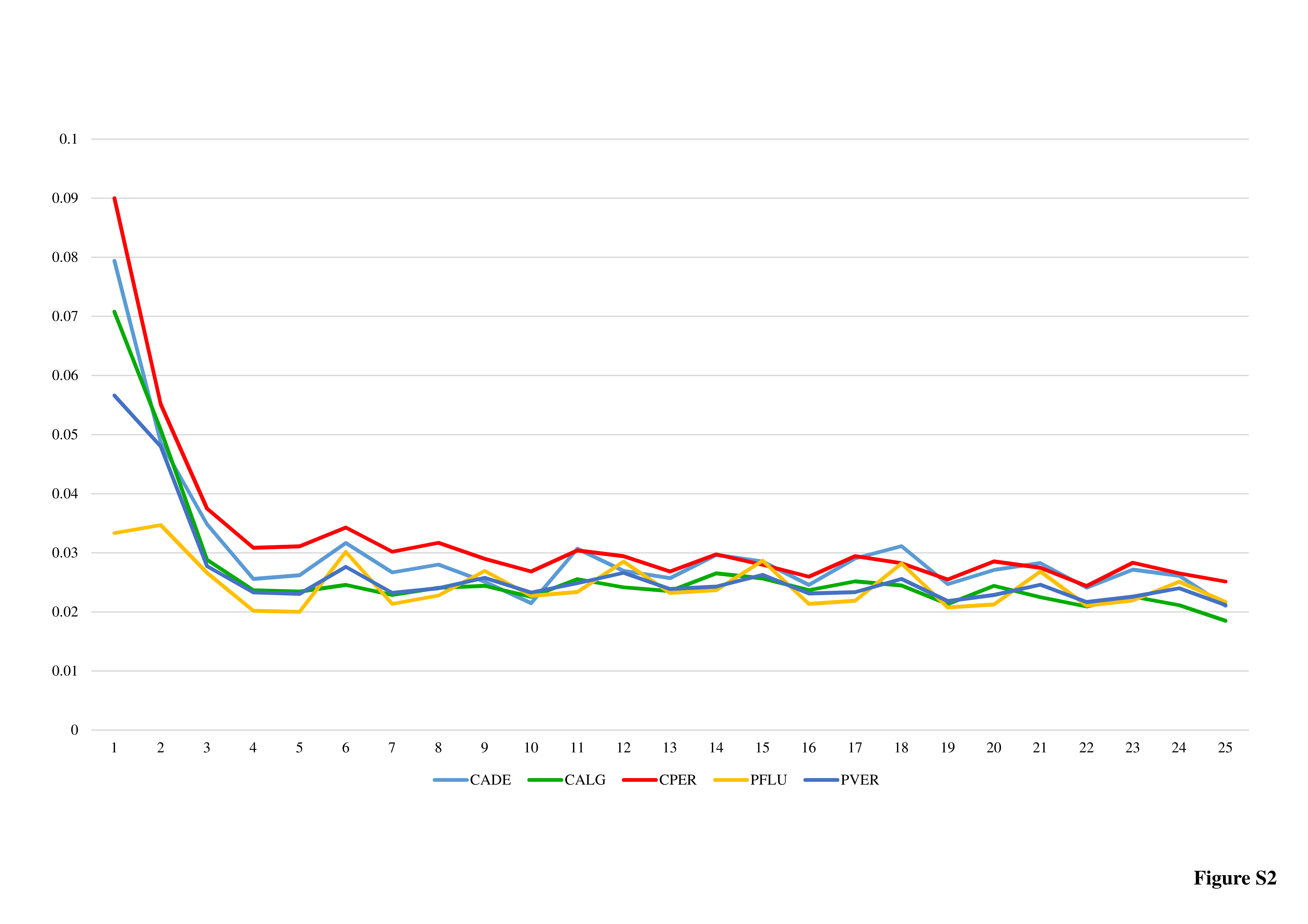

Supplement: Additional file 3: Figure S2. — Cytosine to thymine substitution frequency at the 5′ end of the sequenced reads. The plot displays the cytosine deamination pattern of the Clostridium sp. CADE, C. algidicarnis CALG, C. perfringens CPER, P. fluorescens PFLU, and P. veronii PVER selected reads from the Ötzi’s metagenomic samples. The y axis reports the C to T substitution frequency, while the x axis indicates the distance from the 5′ end of the sequence reads. [file 40168_2016_221_MOESM3_ESM.tif]

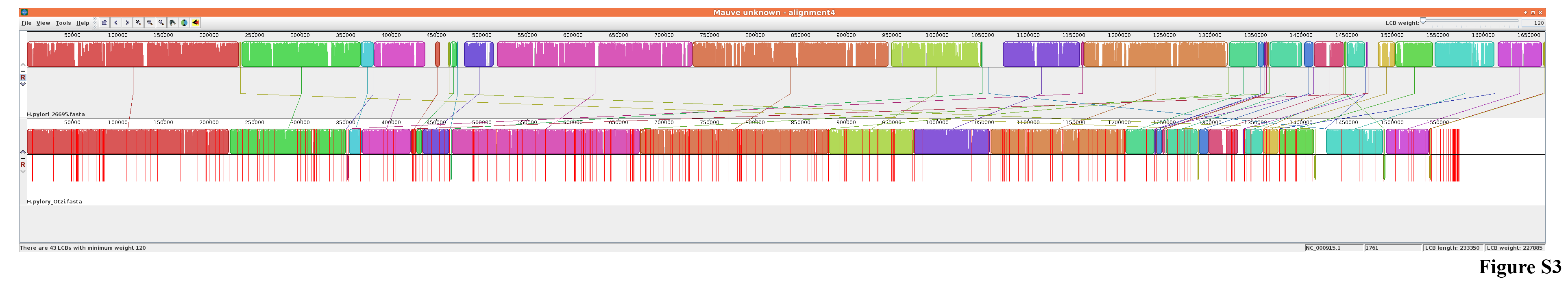

Supplement: Additional file 4: Figure S3. — Mauve alignment of reconstructed ancient H. pylori to the reference H. pylori 26695. On top the reference H. pylori 26695 genome is depicted, while the reordered contigs of the reconstructed ancient H. pylori from the Ötzi’s metagenomics samples is indicated on the bottom part of the figure. Each colored block corresponds to a conserved genomic region between the two genome sequences, while the red line discerns the ancient H. pylori contigs. [file 40168_2016_221_MOESM4_ESM.tif]

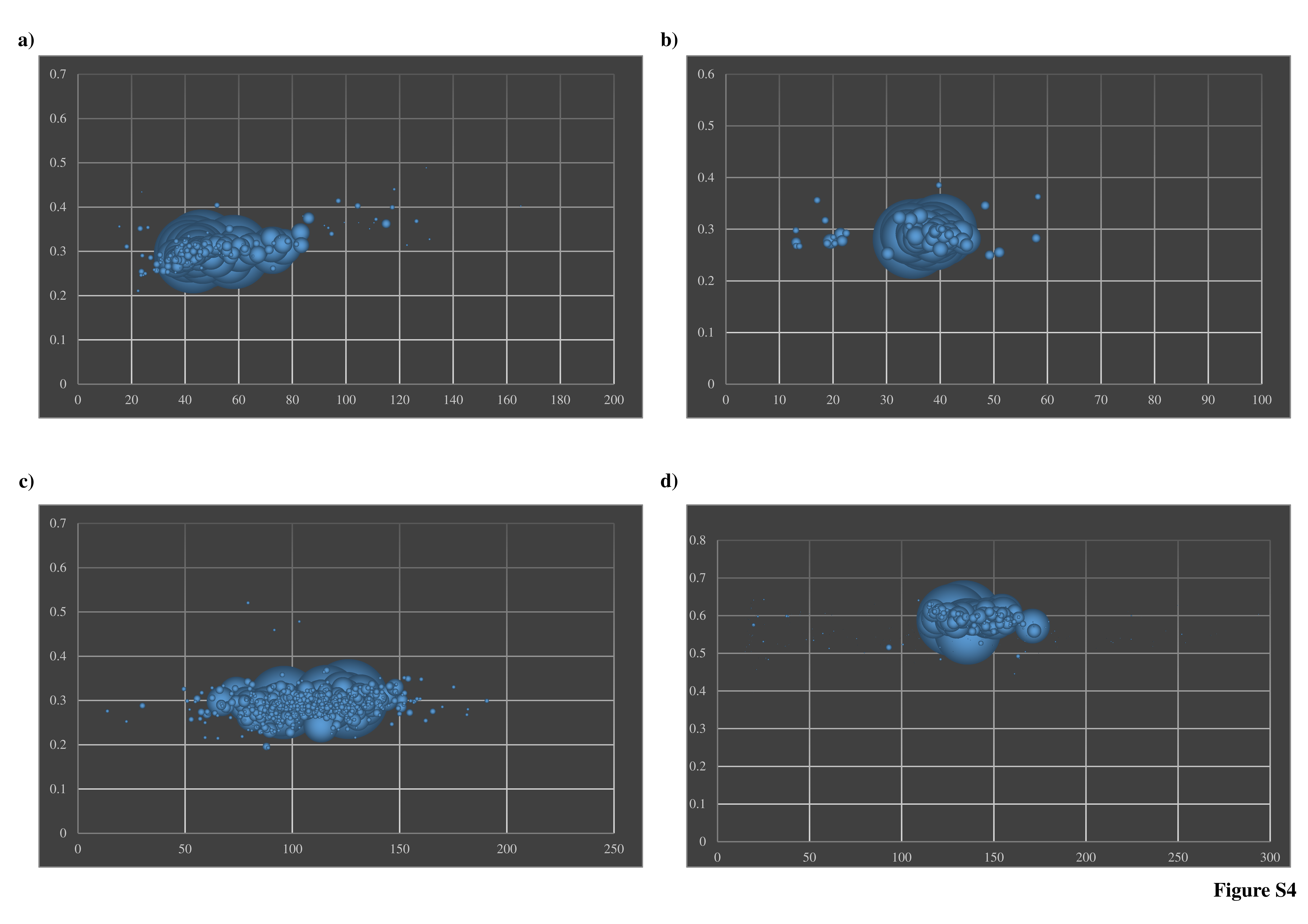

Supplement: Additional file 5: Figure S4. — CG content/coverage plot of ancient reconstructed genome contigs. Panel a displays the CG content distribution of the Clostridium sp. CADE contigs; similarly, panels b to d display this for C. algidicarnis CALG, C. perfringens CPER, and P. veronii PVER contigs, respectively. [file 40168_2016_221_MOESM5_ESM.tif]

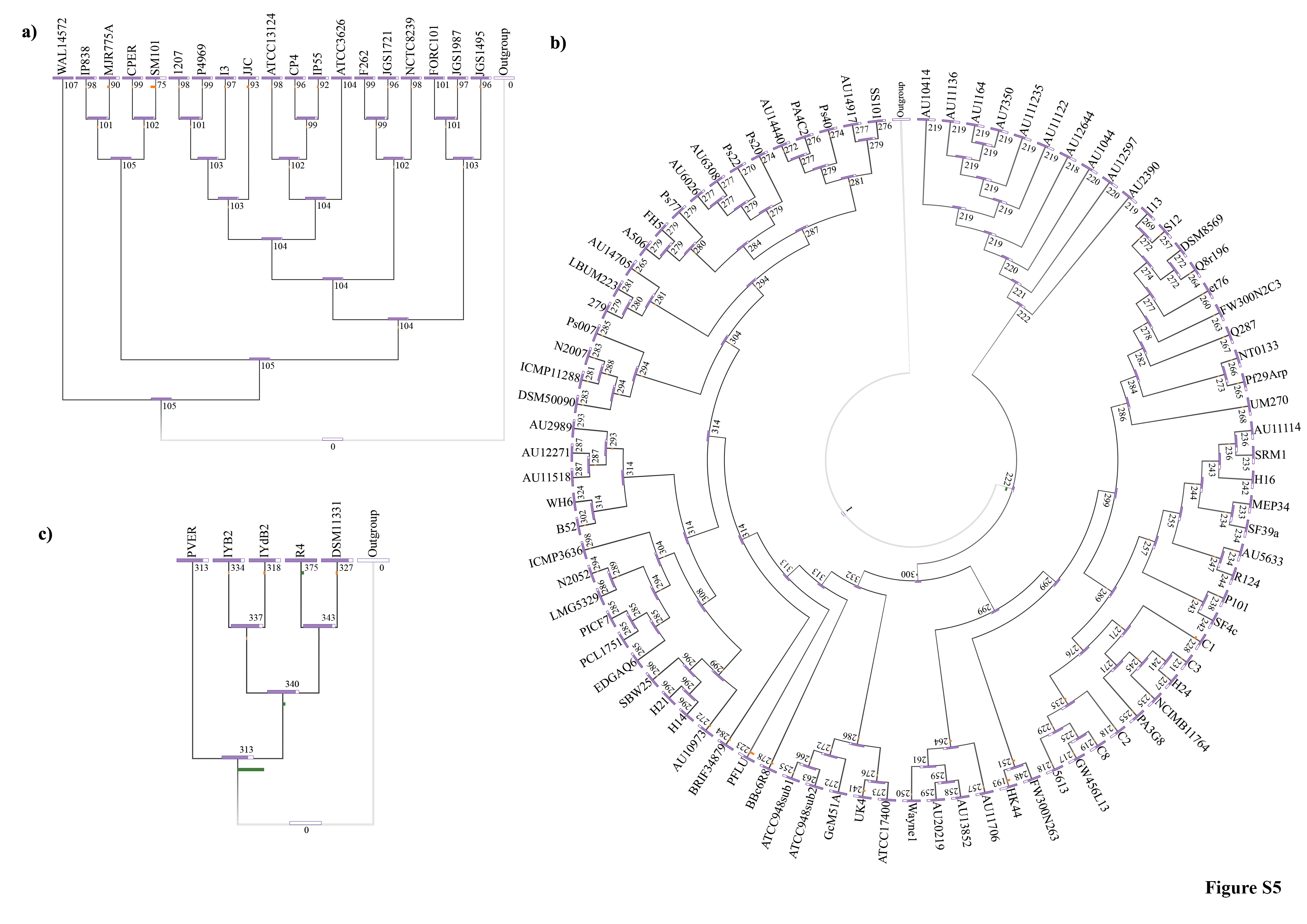

Supplement: Additional file 6: Figure S5. — Evolutionary gain and loss analysis based on predicted virulence factors within C. perfringens, P. fluorescens, and P. veronii species. Panel a displays the core gene-based supertree of C. perfringens strains, where each node reports the number of predicted virulence factor COGs identified for each strain. Furthermore, gains and losses are indicated by green and orange bars on the edge leading to each node. Panels b and c show the same analysis conducted on P. fluorescens and P. veronii species, respectively. [file 40168_2016_221_MOESM6_ESM.tif]

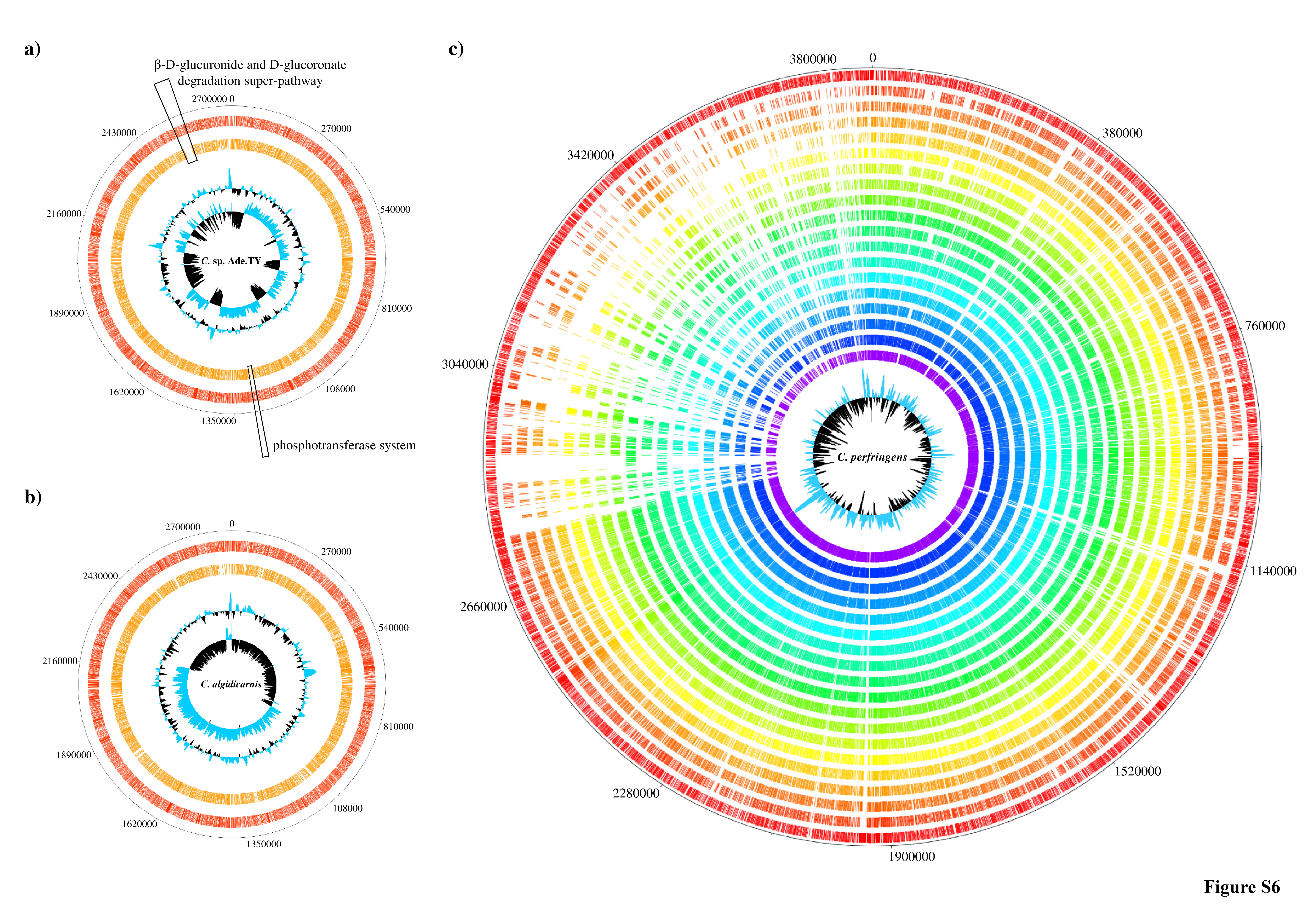

Supplement: Additional file 7: Figure S6. — Comparative genomic analysis of Clostridium sp. CADE, C. algidicarnis CALG, and C. perfringens CPER with other fully sequenced strains. Panel a displays the circular genome atlas of Clostridium sp. CADE (red circle) with mapped orthologues (defined as reciprocal best BLASTp hits with more than 50% identity over at least 50% of both protein lengths) in public available Clostridium sp. Ade.TY genome (orange circle). Internal circles illustrate GC% deviation and GC skew (G − C/G + C). Panel b and c shows the same circular genome atlas of C. algidicarnis CALG and C. perfringens CPER, respectively. [file 40168_2016_221_MOESM7_ESM.jpg]
